# Implementation of a Patient Reported Experience Measure in a Dutch disability care organisation: a qualitative study

**DOI:** 10.1186/s41687-019-0169-3

**Published:** 2020-01-14

**Authors:** Marjolein van Rooijen, Stephanie Lenzen, Ruth Dalemans, Albine Moser, Anna Beurskens

**Affiliations:** 1 Department of Family Medicine, CAPHRI School for Public Health and Primary Care, Maastricht University, P. Debyeplein 1, 6229 HA Maastricht, Netherlands; 2 Research Centre for Autonomy and Participation of Persons with a Chronic Illness, Zuyd University of Applied Sciences, Heerlen, Netherlands

**Keywords:** Patient reported experience measure, Integrated measurement, Consolidated framework for implementation research, Intellectual disabilities, Communication vulnerable, Quality of care

## Abstract

**Background:**

Patient Reported Experience Measures are promoted to be used as an integrated measurement approach in which outcomes are used to improve individual care (micro level), organisational quality (meso level) and external justification (macro level). However, a deeper understanding of implementation issues of these measures is necessary. The narrative Patient Reported Experience Measure “Dit vind ik ervan!” (English “How I feel about it!”) is used in the Dutch disability care sector, but insight into its’ current use is lacking. We aimed to provide insight into experiences with the implementation and current ways of working with “Dit vind ik ervan!” as an integrated measurement strategy.

A descriptive qualitative study was done at a disability care organisation. Data were collected by nine documentations, seven observations, 11 interviews and three focus groups. We applied deductive content analysis using the Consolidated Framework for Implementation Research as a framework.

**Results:**

Our analysis revealed facilitators and barriers for the implementation of “Dit vind ik ervan!”. We found most barriers at the micro level. Professionals and clients appreciated the measure’s narrative approach, but struggled to perform it with communication vulnerable clients. Some clients, professionals and team leaders were unfamiliar with the measure’s aim and benefit. On the meso level, implementation was done top-down, and the management’s vision using the measure as an integrated measurement approach was insufficiently shared throughout the organisation.

**Conclusions:**

Our study shows that Patient Reported Experience Measures have the potential to be used as an integrated measurement strategy. Yet, we found barriers at the micro level, which might have influenced using the measurement outcomes at the meso and macro level. Tailored implementation strategies, mostly focusing on designing and preparing the implementation on the micro level, need to be developed in co-creation with all stakeholders.

## Background

The inclusion of clients’ experiences is a central pillar of quality in health care [13]. Measuring clients’ experiences is thought to provide transparency, to improve client safety and clinical effectiveness, and can lead to more involvement in decision-making and to more effective healthcare professional-client relationships [13, 17, 24]. Measuring experiences can be done with Patient Reported Experience Measures (PREMs), which are defined as “a measure of patients’ (or clients’) perceptions of their personal experiences of the healthcare they have received” [5]. The Dutch Association for People with Disabilities promotes the use of PREMs and judged a selected number of PREMs as suitable for the Dutch disability sector [27]. One of those PREMs is called “Dit vind ik ervan!” (English “How I feel about it!”), which was especially developed for people with intellectual, developmental and acquired disabilities [11, 28]. This group often faces communication vulnerabilities. Communication vulnerable people are people experiencing difficulties with the expression of needs or understanding information [29]. “Dit vind ik ervan!” is a personalized and narrative PREM, consisting of an exploratory dialogue between the professional and the client to discover the client’s personal experiences with care. The PREM includes ten topics: family, feelings, house, feeling safe, friends and acquaintances, participating, help, choosing, doing, and body. Clients themselves decide whether and which topics they want to discuss. Conversation cards, that visualize the topics, are provided to support clients to express their experiences. Clients can score topics from bad to good and indicate whether the topic needs change.

Currently, “Dit vind ik ervan!” is used at over 30 disability organisations in the Netherlands. One of them is the “Stichting Gehandicaptenzorg Limburg” (SGL) (English: ‘Disability Care Foundation Limburg’), which offers supported living and living arrangements to people with severe acquired, intellectual, and developmental disabilities, mostly for people with Acquired Brain Injuries (ABI). At SGL, professionals are trained in two versions of “Dit vind ik ervan!”: (1) “I am speaking”: suitable for clients who can verbally communicate; the dialogue is performed with the client alone, and (2) “I see and am speaking”: suitable for clients who need support to verbally communicate; the dialogue is performed with the client and the client’s informal caregiver [12].

Recently, PREMs are increasingly promoted to be used as an integrated measurement strategy, also in the Dutch disability sector [4, 9]. This means that PREM outcomes are used integrative at three levels: (1) at the micro level, which is the client-professional level, to enhance the delivery of appropriate care and the development of individual support plans: (2) at the meso level, which is the organisational level, to monitor quality of care and enhance team reflection, and (3) at the macro level, to facilitate external reporting about quality of care [4, 9]. However, uncertainty and limited insights exist regarding the implementation of PREMs in routine care for this purpose [9]. Foster’s systematic review shows that time and resources are required to design the PREM process in policy of the organisation (i.e., planning how data will be managed and used) and to prepare the organisation for the implementation (i.e., training professionals to use the PREM) [15]. However, a deeper understanding of practical issues surrounding implementation of PREMs to understand these processes seems to be necessary [3].

The management of SGL also intends to use “Dit vind ik ervan!” as an integrated measurement strategy. Despite implementation efforts, such as training sessions, they think that until now “Dit vind ik ervan!” is not implemented structurally in routine care, nor have the results been used at all three levels (micro, meso, macro). They lack insight into which factors hinder or facilitate the implementation of the PREMs as an integrated measurement strategy.

This study aimed at providing greater insights into experiences with the implementation and current ways of working with the PREM “Dit vind ik ervan!” as an integrated measurement strategy in routine care by answering the following research question:

- What is the state of the art of the implementation of an integrated measurement approach using a PREM in care for clients with acquired brain injuries, from multiple perspectives?

## Methods

### Design

A descriptive qualitative design, using a deductive contents analysis [14], was applied because of its’ potential to describe a poorly understood phenomenon and because it answers questions such as who, what, and where [22]. We investigated experiences with regard to using a PREM as an integrated measurement approach within SGL by incorporating key-stakeholders involved in the process of working with the PREM on multiple levels. We used the Consolidated Framework for Implementation Research (CFIR) as guidance for data collection and analysis [8]. The CFIR describes 39 sub-domains divided across five main domains: (1) Intervention Characteristics (key attributes of the intervention that can influence implementation), (2) Outer Setting (external factors that can influence implementation), (3) Inner Setting (internal (organisational) factors that can influence implementation), (4) Characteristics of Individuals (characteristics of individuals which can influence implementation (healthcare professionals and clients)), (5) Implementation Process (process factors that can influence implementation) [8].

### Data collection and participants

Data were collected from September 2017 until July 2018 by means of documents, observations, interviews, and focus groups, providing perspectives from micro, meso and macro level. The data collection focus was defined at three decision points, after analysing: (1) documentation and observations, (2) interviews and (3) focus groups. Table 1 provides an overview of all collected data.

**Table 1:** Intervention Characteristics (IC), Outer Setting (OS), Inner Setting (IS), Characteristics of Individuals (CoI), Implementation Process (IP)

Data sources	Domains of CFIR
Documents (*n* = 9, 99 pages total)	
Document about requirements for the quality report for the Dutch Association of Healthcare Providers for People with Disabilities, based on the quality framework (13 pages)	OS
Document with principle assumptions of “Dit vind ik ervan!” (1 pages)	IC
General brochure about “Dit vind ik ervan!” (3 pages)	IC
Attributes brochure about “Dit vind ik ervan!” (2 pages)	IC
Feedback report of the reflection visitation at SGL of the foundation of “Dit vind ik ervan!” (4 pages)	OS, IS, IP
Internal audit summary on clients’ and professionals’ experiences with the PREM performed by SGL (6 pages)	IS, IP
Internal audit report of 5 interviews with clients about the “Dit vind ik ervan!” procedure (26 pages)	IS, IP
Internal audit report of 6 interviews with professionals about the “Dit vind ik ervan!” procedure (35 pages)	IC, IS, CoI, IP
Analysis report random sample survey of electronic client records of the “Dit vind ik ervan!” procedure and action points resulting from it (10 pages)	CoI, IP
Observations (*n* = 7)	
2 PREM training sessions for SGL team leaders provided by an external trainer employed by the “Dit vind ik ervan!” foundation (10 team leaders and one trainer) (duration 3 h each)	IS, CoI, IP
Auditors instruction session for SGL internal audit interviews (2 team leaders, 2 professionals, a secretary and a quality manager of SGL) (duration 30 min)	IS, CoI
Observation of 2 internal audit interviews with clients (duration 20–42 min)	CoI, IP
Observation of 2 internal audit interviews with professionals (duration 20–42 min)	IS, CoI
Interviews (*n* = 11)	
8 clients (4F, 4 M) aged 35–66 years, living at SGL between 5 and 36 years (duration 24–50 min) Clients’ communication vulnerability tested using communication-vulnerability screening lists, developed by Zuyd University of Applied Sciences [23].	CoI, IP
Interview with the SGL general manager (F), age 53 years, working at SGL for 11 years (duration 70 min)	IC, OS, IS, CoI, IP
Interview with a researcher (M), age 37 years, working for the “Dit vind ik ervan!” foundation 3 years (duration 80 min)	IC, OS
Interview with a manager (F), age 37 years, working at the “Dit vind ik ervan!” foundation 3 years (duration 77 min)	IC, OS
Focus groups (*n* = 3)	
2 Focus groups with 3 (3F) and with 4 (1 M, 3F) professionals. Aged 22 – 53 years, and working at SGL 0.5–12 years (duration 35–60 min)	IC, IS, CoI, IP
Focus group with a quality advisor and 2 PREM trainers of SGL (3F). Aged 44–50 years, and working at SGL 11–17 years (duration 93 min)	IC, OS, IS, CoI, IP

#### Documents

Nine documents were purposively sampled to explore SGL’s working routines with the PREM and instructions provided to work with the PREM and instructions to create the quality report in the Dutch disability sector. Documents were provided by SGL, by the foundation that developed the PREM or they were openly available online, published after 2016.

#### Observations

Seven observation events (e.g., team leaders’ PREM-trainings), were purposively sampled to study team leaders’, healthcare professionals’ and clients’ knowledge and behaviour with regard to using the PREM. We made observations and took field notes.

#### Interviews

Individual semi-structured interviews with clients (*n* = 8), the manager of SGL (*n* = 1) and the manager and researcher appointed by the PREM foundation (*n* = 2) were conducted. All respondents were purposively sampled. Clients were approached by team leaders of SGL to get insight into experiences with using “Dit vind ik ervan!”. We included clients who (a) had been interviewed with “Dit vind ik ervan!” within the last 3 months, (b) lived at SGL-supported living facilities, and (c) were able to communicate about their experiences. Clients with communicative and cognitive disabilities were facilitated as much as possible for example by interviewing in quite environments, using conversation cards and by providing enough time. The interview with the SGL manager provided insights into the decision process and considerations regarding the implementation of “Dit vind ik ervan!”. The manager and researcher of the PREM foundation provided insights in the development and experiences with the PREM within other organisations.

#### Focus groups

We purposively sampled participants for three focus groups. In the first focus group we interviewed one quality manager and two trainers, to gather insights about the PREM training and work procedures, but also about rationales of decisions made regarding the PREM implementation. The other two focus groups included healthcare professionals who had been trained to perform the PREM, to gather recent experiences with using “Dit vind ik ervan!” in routine care.

### Data analysis

All interviews and focus groups were audiotaped and transcribed verbatim. Then, deductive content analysis, using CFIR as a framework, was applied to all field notes, observational notes and transcripts. The analysis consisted of three phases: preparation, organising and reporting [14]. In the preparation phase, we submerged ourselves in the data by reading and re-reading all data transcripts. Next, we selected units of analysis based on relevance to the research question. One unit of analysis consisted mostly of more than one sentence to justify the context-rich data. From this, we selected units of meaning that contributed to answering the research question. In the organising phase, two researchers (MvR and SL) first deductively developed a structured analysis matrix based on the five main domains of CFIR. This matrix was pre-tested with the first data sources and refined where necessary. Then data were reviewed multiple times and open coding was done. Codes were inductively grouped into categories forming main- and some sub-categories within the five domains of the CFIR [14]. We used the Nvivo 12 software to support the analysis. In the reporting phase, we described (sub)categories.

### Ethics

All participants received written and verbal information in advance, gave verbal informed consent prior to the observations and gave written informed consent prior to the interviews and focus groups. Data were treated as confidential and anonymous by code-numbering the data. The study was reviewed by the ethics committee Zuyderland-Zuyd (METCZ20180062).

### Trustworthiness

Trustworthiness was safeguarded applying strategies regarding credibility and transferability [19]. Credibility was enhanced by persistent observation of the data using an iterative strategy of performing and analysing qualitative data (e.g., developing and working with the analysis matrix), thereby studying characteristics of data extensively [14]. Additionally, investigator triangulation took place by analysing sessions with an inter-professional research team consisting of a professor/physiotherapist, a senior researcher/nurse, a post-doc researcher/occupational therapist and a health scientist. Data triangulation was done using multiple data sources (documents, observations, interviews and focus groups) from key-stakeholders on multiple levels. Additionally, data and results were member-checked for accuracy and resonance. With clients, we personally reflected upon their interviews using summaries during a visit, 4 weeks after their interview. With other interview and focus group participants we reflected upon summaries on which they provided feedback via e-mail [6]. To enhance transferability, we provided rich data about the setting, sample, data collection and data analysis procedures [19].

## Results

**Fig. 1:**
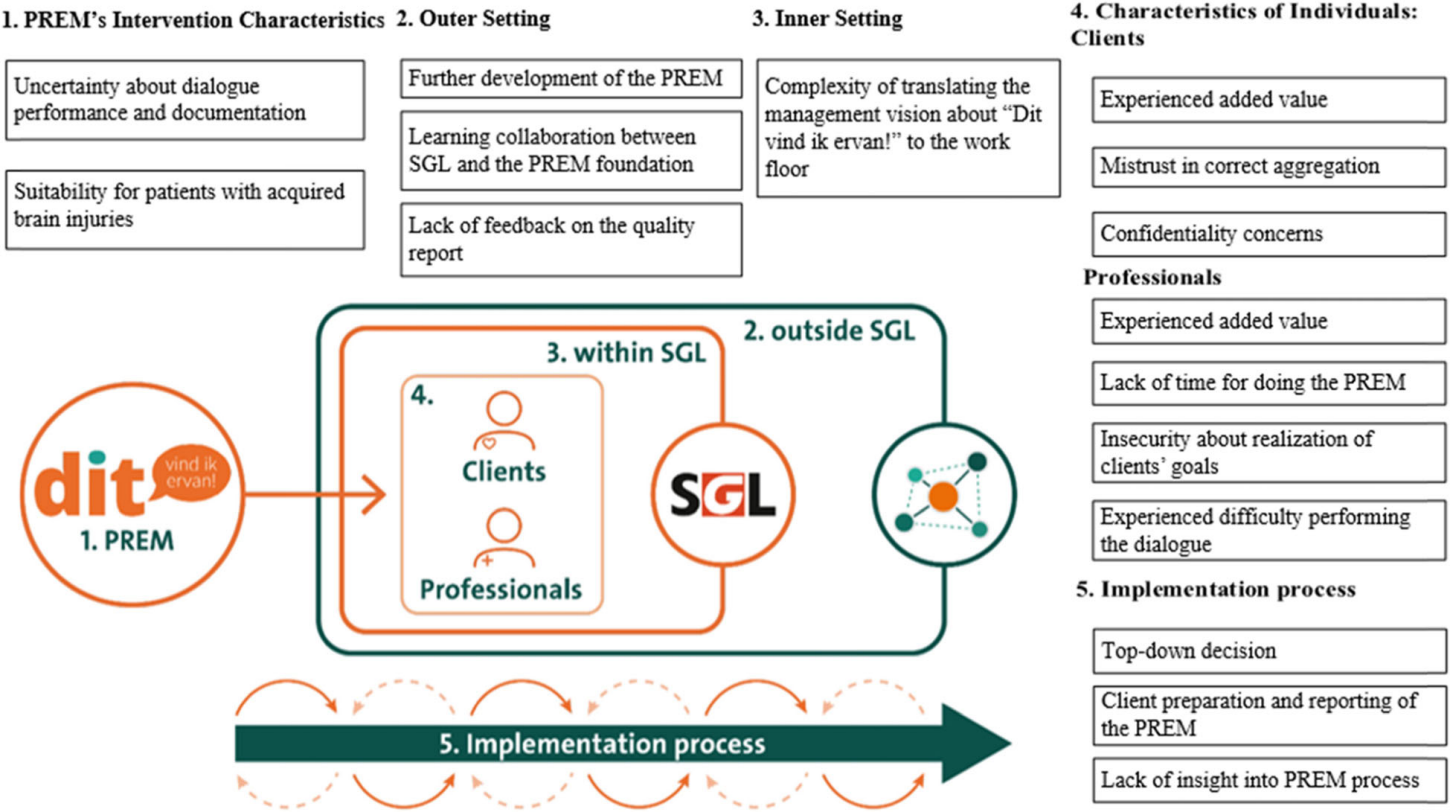
CFIR used for representation of the findings [8]

Our analysis resulted into different topics according to the five CFIR domains, as shown in Fig. 1.

### PREM’s intervention characteristics

This domain focuses on key attributes of the PREM itself, which influence implementation.

#### Uncertainty about dialogue performance and documentation

The exploratory dialogue, the core characteristic of “Dit vind ik ervan!”, is thought to enable clients in sharing their personal experiences. During a two-day training professionals learned to perform the dialogue. However, professionals reported missing clear guidance on how a dialogue of sufficient quality can be performed.

“We have had the training and that was it. You never know if you are doing it good or bad, since at the same time you are performing the dialogue.” (professional SGL)

According to the general brochure of “Dit vind ik ervan!” the dialogue might take place at once, but it can also be done at multiple moments if the client wishes. This openness led to professionals struggling to plan the dialogue. They were also uncertain about when to complete the PREM documentation in the Electronic Client Dossier.

#### Suitability for people with acquired brain injuries

The manager and researcher of “Dit vind ik ervan!” explained that the PREM was developed by five disability organisations who aimed at developing an experience measurement tool specifically for people with communication and cognitive challenges. Nevertheless, during the PREM development little attention was paid to the use of communication supporting tools and strategies for the exploratory dialogue and to creating communication friendly physical environments. Yet, the exploratory dialogue, presented the greatest challenge to communication vulnerable clients, especially to people with ABI. The PREM organisation therefore encourages professionals to use tools and strategies they are already familiar with and use in their daily communication with their clients.

““Dit vind ik ervan!” has the ambition to focus on the dialogue, to connect and use existing communication tools that have already been developed. Though it is, at the moment, not developed by “Dit vind ik ervan!” and maybe not used.” (researcher for “Dit vind ik ervan!”)

### Outer setting

The outer setting domain focuses on external factors that can influence the implementation.

#### Further development of the PREM

The Dutch Association for People with Disabilities demanded scientific research about “Dit vind ik ervan!” on three topics: (1) identification of criteria that are necessary to safeguard the quality of the dialogue, (2) identification of how “Dit vind ik ervan!” contributes to appropriate care and individual support plans for clients, and (3) identification of how “Dit vind ik ervan!” contributes to the legitimacy of and adds value to aggregation of outcomes on multiple levels. Working on these topics was a precondition to become legislated by the Dutch disability sector. Next to legislation, the PREM foundation also believed that it is important to develop instructions for good quality dialogues. They therefore appointed an external researcher to investigate how validity of the PREM can be improved.

“We have to find out how we can scientifically prove the validity of “Dit vind ik ervan!” and with the findings we can improve guidelines for the “Dit vind ik ervan!” users on how the dialogue should take place, what distinguished its quality and which checks we need to build into the process. Thereby improving quality of the conversation as well.” (researcher for “Dit vind ik ervan!”)

#### Learning collaboration between SGL and PREM foundation

SGL trainers, i.e. professionals working at SGL who are trained to train other professionals in working with ‘Dit vind ik ervan!”, and the quality manager were in close contact with the foundation of “Dit vind ik ervan!” by attending yearly meetings and trainers’ sessions, and having regular phone calls and e-mail contact. This collaboration still provides SGL insights in strategies to engage their professionals in the process of implementing “Dit vind ik ervan!”. At the same time, other organisations working with the PREM and the PREM foundation learn from SGL’s experiences.

“I visit them (“Dit vind ik ervan!” meetings) to find answers or inspiration, for example about how you keep people in the organisations involved with “Dit vind ik ervan!” and prevent the employees to sit back and relax after the “Dit vind ik ervan!” trainings took place instead of.’ (quality advisor for SGL)

#### Lack of feedback on the quality report

The Dutch Association for People with Disabilities provided organisations in the disability sector with global guidelines for the creation of the external quality report. The global guidelines included the following aspects: clients’ experiences with quality of being, clients’ experiences with quality of care and a clear compilation of insights from recent quality research. Although SGL used these global guidelines to design their first external quality report, they were unsure about the quality of their report. They explained that they were not always sure about what was expected of them because they had not received feedback on the last report.

“Not really no, at least not that I know. We are told to meet with the guidelines of the quality framework that is what they demand from us. And one of the building blocks of this quality framework is the research into clients experiences and yes that is how we should meet with their guidelines.” (quality advisor for SGL)

### Inner setting

The inner setting focuses on internal (organisational) factors that can influence the implementation.

#### Complexity of translating the management vision about “Dit vind ik ervan!” to the work floor

According to SGL’s trainers, quality manager and general manager “Dit vind ik ervan!” must become a ‘way of life’ within SGL’s culture. The core value of SGL’s vision, i.e. person-centred care, is reflected in “Dit vind ik ervan!”. Despite this management vision, not all team leaders seemed to perceive “Dit vind ik ervan!” as a way to work person-centred. During the PREM training, some team leaders explained that they did not see added value, did not know their role within the PREM performance nor understood its’ goal. This poor embedment among some team leaders might have influenced the uptake and attitude towards the PREM among the professionals. Professionals experienced the PREM as an obligation; they did not discuss outcomes among peers and felt little support from their team leaders.

“She (team leader) says once a year that we need to finish “Dit vind ik ervan!”. “This one is not complete, please fill it out for me”, she says. That’s it! She is not really involved in “Dit vind ik ervan!” herself and that’s not really supportive to us as a team.” (professional SGL)

### Characteristics of individuals

The domain “characteristics of individuals” focuses on individuals, both clients and professionals, affected by the implementation of the PREM.

#### Clients

##### Experienced added value

Clients’ views towards “Dit vind ik ervan!” varied. Some clients liked that they could decide which topics to discuss and felt the PREM helped them in formulating personal care needs and goals.

“The themes we discussed facilitated me in telling my story. And I could also decide what I did and did not want to talk about. And I told her (healthcare professional) what I wanted to become a point of action or what I just wanted to talk about.” (client SGL)

Other clients experienced redundancy in PREM themes and questions they answered for their care plan. They missed a distinguishing factor in the PREM compared to the care plan.

##### Mistrust in correct aggregation

Additionally, some clients were unsure what was done with the PREM outcomes and therefore did not want to participate in “Dit vind ik ervan!” after the first time they participated. One client expressed his mistrust in the translation of his story towards the management and in the external quality report.

“I am afraid my story is translated differently to the big bosses. I think the organisation might interpret my words differently, without providing proper explanation. They can do anything with my words.” (client SGL)

#### Confidentiality concerns

Moreover, one client felt that SGL did not deal the PREM outcomes with confidentiality and anonymity. In his case, an external professional performed the PREM and he felt unsecure if his words were translated correctly and did not feel comfortable that his words were passed on so many times. In his case, his own personal professional was not trained to use the PREM. His outcomes were then discussed with his personal professional and other professionals.

“She (the external professional) sent it (the “Dit vind ik ervan!” report) to my personal professional, and then someone else worked on it as well. So at the end my story has been passed on so many times that, no one will know what I really said.” (client SGL)

### Professionals

#### Experienced added value

Some professionals experienced the PREM training as valuable, as they learned to listen more carefully to clients and to not fill in their clients’ words.

“Maybe to open up more during a conversation and trying to listen more, instead of constantly asking, trying to let the client tell its own story.” (professional SGL)

At the same time, professionals expressed that they felt that “Dit vind ik ervan!” was redundant, as they did not see the distinguishing factors between the PREM, the client care plan and other methods used at SGL. Moreover, professionals felt that “Dit vind ik ervan!” was not necessary for person-centred work; they had the idea that they already used a person-centred approach. They felt that they already knew their clients’ wishes because they had already known their clients for a long time. They experienced a good relationship with their clients and thought that no exploratory dialogue was needed to have conversations with clients.

“Because that is what we tell our clients … If you have something on your mind, just come to us, tell us your problem and we will deal with it. We do not need “Dit vind ik ervan!” to do that!” (professional SGL)

#### Lack of time for doing the PREM

Professionals also felt that using and documenting the PREM took a lot of time. It was challenging for them to prioritize the PREM within daily-care responsibilities.

“I have not had time for that yet (answering the question when of the professional took care of reporting the PREM). This is because we are understaffed at the moment, therefore we only have time for daily care and little time to sit behind a computer.” (professional SGL)

#### Insecurity about realization of clients’ goals

“It is really a pity if someone expresses a wish which you can’t grant because the client needs to be around a professional who is able to suck the client out.” (trainer SGL)

During focus groups, professionals explained that they worried about the realisation of their clients’ wishes. A trainer said she faced problems with resources such as lack of time and skilled staff when clients with special medical needs expressed wishes she could not grant.

#### Experienced difficulty performing the dialogue

Additionally, professionals experienced challenges with performing the dialogue with communication-vulnerable clients and with clients with cognitive disabilities. Professionals struggled to adapt the dialogue to the clients’ communicational levels, did not know how to use communicative supportive tools and strategies, and therefore they often just did not perform “Dit vind ik ervan!” with some clients.

“I have a client and she cannot do anything. She has a type of MS, so verbally she lost everything. And cognitively she cannot do anything either, so there is nothing you can do with “Dit vind ik ervan!”. Everything comes from us.” (professional SGL)

### Implementation process

The domain implementation process within CFIR discusses process factors that can influence implementation.

#### Top-down decision

The manager of SGL explained that the board chose “Dit vind ik ervan!” as a PREM since it had the ability to discover clients’ personal goals, which fitted within SGL’s vision. Additionally, SGL’s central client advisory committee was consulted before the implementation started. The committee raised objections against the confidentiality and anonymity of the PREMs outcomes. Yet, they gave their permission to start working with it. Managers, team leaders, professionals and clients were not consulted before the implementation started, and no pilots were performed due to time pressure on the quality report. However, a kick-off event was organised to engage managers, team leaders, and some professionals with the PREM.

“We were not consulted during the decision-making process for the instrument, no. At the time of our training session, the decision had already been made.” (trainer for SGL)

#### Client preparation and reporting of the PREM

The internal audit showed that professionals did not always offer clients the option to prepare themselves. Some clients said they liked the opportunity to think about the topics beforehand, whereas others found the idea of preparation confusing.

Moreover, after the dialogue took place, the outcomes of “Dit vind ik ervan!” should have been member-checked with clients, and professionals told us they mostly did. Nevertheless, when asked during the interviews, none of the clients were able to hand over a PREM-report, and some were unsure if they had received it.

“It sometimes just does not happen. We often lack time and are happy if we get all clients taken care of. We do not have time to go sit behind the computer. Reporting about daily practice, that is what we do.” (professional SGL)

#### Lack of insights into the PREM process

The management of SGL and trainers explained that they lacked insights into how the dialogue was performed. Although they could control the number of performed PREMs in the Electronic Client Dossiers, this did not represent the quality of the performed dialogues.

“We can check Pluriform (Electronic Client Dossier) conversations lists’ and how those look, but I think it is mainly team leaders who play the essential role in that process.” (quality advisor for SGL)

## Discussion

This study aimed at providing greater insights into experiences with the implementation and current ways of working with the PREM “Dit vind ik ervan!” as an integrated measurement strategy in routine care. We found factors facilitating and/or hindering the implementation of the PREM on the micro level (clients-professional level; relating to the CFIR domains ‘Intervention Characteristics’, ‘Characteristics of Individuals’ and ‘Implementation Process’), the meso level (within the management in and between SGL locations; relating to the CFIR domains ‘Inner Setting’ and ‘Implementation Process’), and the macro level (between the SGL management and health insurance offices; relating to the CFIR domains ‘Outer Setting’ and ‘Implementation Process’) (Fig. 1).

On the micro level, we found the exploratory dialogue of the PREM to be complex. Although the PREM was developed specifically for people with intellectual disabilities and for this reason is personalized and narrative, it is especially this exploratory dialogue that complicates the use of the PREM with people with ABIs. Professionals in our study struggled with facilitating clients in expressing their experiences and not filling in their sentences. Additionally, they were unfamiliar with other communication supportive tools or strategies. Our findings confirm earlier research showing that clients are often insufficiently supported to express themselves during a dialogue with a professional and at times face difficulties understanding professionals [23]. Communication-supportive tools and strategies might need to focus on three levels: (1) professionals’ education, skills and attitudes towards communication-supportive tools and strategies, (2) preparing the conversation including a communication friendly environment and (3) the use of ad hoc augmentative and alternative communication such as picto-cards, writing and drawing [23]. These tools and strategies can also be used within the implementation of “Dit vind ik ervan!” and other narrative PREMs.

Moreover, it was striking that clients in our study reported not feeling safe to share their experiences with professionals, whereas professionals felt their care relationship was good. Literature shows that a precondition for a good PREM performance is a safe care relationship [21, 26]. Therefore, factors positively influencing the care relationship should be addressed before the PREM is implemented [13, 24]. This can be done by offering programs focussing on the improvement of the client-professional relationship, in which professionals improve their client-specific communication skills such as listening to a client’s voice, non-verbal behaviour, and basic empathy. Other options are teaching professionals to offer their clients the option of doing the PREM with a professional of their choice, as well as being clear about what happens with their PREM data [10, 16].

Furthermore, clients, professionals and team leaders were not always familiar with the aim of the PREM and experienced redundancy with other instruments used at SGL. The distinction between discussing a client’s individual care plan and performing the PREM dialogue was unclear to clients, professionals and team leaders. The literature shows that a crucial element in the implementation of PREMs is the organisation’s investment (time and resources) in designing the PREM process (i.e., thinking about how the PREM data will be used to improve care at the micro-level, how data will be managed and how the PREM fits into routine care) [15]. The fact that participants were unsure about the PREM’s added value reveals that they were insufficiently involved or informed about how to use the data and how this supports client centred care.

This was also seen at the meso level. SGL’s management vision to use the PREM as an integrated measurement strategy and as a way to provide personalized care was insufficiently shared throughout the organisation. Some professionals experienced a lack of their team leaders’ support, which negatively relates to professionals’ enthusiasm and adaptation of skills to use PREMs [18]. Consequently, the use and outcomes of the PREM were rarely discussed during team reflection meetings. Indeed, a supportive manager’s attitude is essential for stronger implementation climate and implementation effectiveness [9, 20]. The engagement of team-leaders is important to promote organisations goals, to explain the instruments priority and to negotiate for resources [1, 7].

In addition, the top-down implementation approach in which only management and the client advisory board were consulted might have complicated implementation. Research shows that the implementation of PREMs needs to be prepared thoroughly (e.g. by training professionals and convincing them of the value of the PREM) [15]. Implementation success is also positively influenced by spreading decision-making power within the organisation and performing needs assessments prior to implementation [2]. Therefore stakeholders from all levels within the organisation should be involved to create strategies to improve the PREM implementation.

On the macro level, we found that SGL management missed feedback and concrete guidelines from health insurance organisations to create the quality report. As the PREM is personalized and narrative, aggregating qualitative data is more time-consuming and difficult compared to quantitative PREMs. Providing feedback from the macro level is found to clarify reporting needs and potentially increasing managements’ engagement in writing the quality report [25].

In general, it became clear that most barriers were found on the micro level (e.g., difficulties to perform the dialogue and lack of experienced added value) which potentially influence the quality and usefulness of PREM outcomes on the meso and macro level. Our study thereby confirms earlier research about implementation of PREMs, that highlight the importance of designing the PREM process and thoroughly preparing the implementation of the PREM in order to support stakeholders to be ready to use the PREM and its outcomes in daily practice [15].

### Strengths and limitations

A strength of our study is the use of the CFIR, which we used to gather and analyse data from multiple perspectives; the clients, professionals, managers and PREM developing foundation, on all three levels (micro, meso, macro). The CFIR domains guided the researchers’ focus, starting with observations and documents from a broader spectrum, becoming more specific in the later performed interviews and focus groups. Another strength is that we involved clients on several levels by consulting SGL’s client advisory board to ask for feedback on the research question and by interviewing clients about their experiences. Moreover, trustworthiness was established by many different strategies: member-checking, peer-reviewing the analysis and results, and methodological data- and investigator-triangulation. However, this study is also subject to limitations. First, despite the team leaders’ central role in using the PREM, team leaders were not interviewed individually. Yet, we gathered data by observations and we held focus groups in two locations with two different team leaders involved. Second, PREM performance was not directly observed, since we expected that our presence might influence the performance and the clients’ feelings of safety in the conversation. However, we did interview professionals and clients about how the PREM was used and their experiences.

## Conclusion

In depth insights in the implementation and current way of working show that personalized and narrative PREMs such as “Dit vind ik ervan!” have the potential to be used as an integrated measurement strategy. We found several barriers with the use of such a PREM at the micro level (e.g. complexity to perform the exploratory dialogue with the client-group, client’ unsafe feelings of telling their stories, client and professionals not experiencing an added value in comparison with other instruments used). To implement the PREM as an integrated measurement strategy, tailored implementation strategies, mostly focusing on designing and preparing the implementation on the micro level (e.g. preparing client, designing the process of data use in daily practice, facilitating administration and training professionals), need to be developed in co-creation with all-important stakeholders.

## Data Availability

The written informed consents signed by all participants authorizing the publication of research data are safely stored at Maastricht University by of the corresponding author, available to the Editor at any time.

## Abbreviations

DVIE: ‘Dit vind ik ervan!’ (English ‘How I feel about it!’)
ABI: Acquired Brain Injuries
CFIR: Consolidated Framework for Implementation Research
PREM: Patient Reported Experience Measure
SGL: Stichting Gehandicaptenzorg Limburg (English “Disability Care Foundation Limburg”)
